# TCRβ clones in muscle tissue share structural features in patients with idiopathic inflammatory myopathy and are associated with disease activity

**DOI:** 10.3389/fimmu.2023.1279055

**Published:** 2024-01-10

**Authors:** Dornatien C. Anang, Hannah A. W. Walter, Johan Lim, Ilse T. G. Niewold, Linda van der Weele, Eleonora Aronica, Filip Eftimov, Joost Raaphorst, Barbera D. C. van Schaik, Antoine H. C. van Kampen, Anneke J. van der Kooi, Niek de Vries

**Affiliations:** ^1^ Department of Rheumatology and Clinical Immunology, Amsterdam Rheumatology and Immunology Center, Amsterdam UMC, University of Amsterdam, Amsterdam, Netherlands; ^2^ Department of Experimental Immunology, Amsterdam Infection and Immunity Institute, Amsterdam UMC, University of Amsterdam, Amsterdam, Netherlands; ^3^ Department of Genome Analysis, Amsterdam UMC, University of Amsterdam, Amsterdam, Netherlands; ^4^ Department of Neurology, Amsterdam UMC, University of Amsterdam, Amsterdam Neuroscience, Amsterdam, Netherlands; ^5^ Department of (Neuro) Pathology, Amsterdam Neuroscience, Amsterdam UMC, University of Amsterdam, Amsterdam, Netherlands; ^6^ Bioinformatics Laboratory, Department of Epidemiology and Data Science, Amsterdam Public Health Institute, Amsterdam Infection and Immunity Institute, Amsterdam UMC, University of Amsterdam, Amsterdam, Netherlands

**Keywords:** myositis, T cells, AIRR seq, T cell receptor (TCR), adaptive immunity

## Abstract

**Objectives:**

To characterize the T cell receptor (TCRβ) repertoire in peripheral blood and muscle tissues of treatment naïve patients with newly diagnosed idiopathic inflammatory myopathies (IIMs).

**Methods:**

High throughput RNA sequencing of the TCRβ chain was performed in peripheral blood and muscle tissue in twenty newly-diagnosed treatment-naïve IIM patients (9 DM, 5 NM/OM, 5 IMNM and 1 ASyS) and healthy controls. Results thereof were correlated with markers of disease activity.

**Results:**

Muscle tissue of IIM patients shows more expansion of TCRβ clones and decreased diversity when compared to peripheral blood of IIM as well as healthy controls (both p=0.0001). Several expanded TCRβ clones in muscle are tissue restricted and cannot be retrieved in peripheral blood. These clones have significantly longer CDR3 regions when compared to clones (also) found in circulation (p=0.0002), while their CDR3 region is more hydrophobic (p<0.01). Network analysis shows that clonal TCRβ signatures are shared between patients. Increased clonal expansion in muscle tissue is significantly correlated with increased CK levels (p=0.03), while it tends to correlate with decreased muscle strength (p=0.08).

**Conclusion:**

Network analysis of clones in muscle of IIM patients shows shared clusters of sequences across patients. Muscle-restricted CDR3 TCRβ clones show specific structural features in their T cell receptor. Our results **indicate** that clonal TCRβ expansion in muscle tissue might be associated with disease activity. Collectively, these findings support a role for specific clonal T cell responses in muscle tissue in the pathogenesis of the IIM subtypes studied.

## Introduction

Subacute proximal muscle weakness is a characteristic feature of idiopathic inflammatory myopathies (IIM). The main subtypes are dermatomyositis (DM), antisynthetase syndrome (ASyS), immune-mediated necrotizing myopathy (IMNM), non-specific/overlap myositis (NM/OM), polymyositis (PM) and inclusion body myositis (IBM) (1, 2). Despite therapy, most patients (70%) have a chronic or polyphasic disease course and develop significant residual disability and reduced quality of life (3). Consequently, there is a clear need for a better understanding of its disease pathogenesis to identify novel therapeutic targets.

For decades, lymphocytes have been implicated in the mechanisms responsible for the development of IIMs. For B-lymphocytes this view is supported by the presence of plasma cells in muscle biopsies (4, 5), the presence of myositis specific and myositis related antibodies in blood in 60 to 70% of the IIM patients (6), the effectiveness of B cell directed therapies (7, 8), and the presence of dominant B-cell receptor clones in muscle tissue and peripheral blood which correlates with better response to intravenous immunoglobulins (9).

Besides B cells, also T cells of different lineages have been implicated in the pathogenesis of IIM patients. Cytotoxic T cells, which often produce toxic granules such as granzymes and perforins, are present in muscle tissues of PM, juvenile DM and IBM patients (10–12) In addition, expanded T cell clones with cytotoxic properties have been reported in DM and IMNM patients (13). Several reports describe skewing of various T cells subsets in peripheral blood, including subsets which provide help to B cells (14–16). The latter is not unexpected, given the key role these cells play in the generation of serological responses. Few studies have explored the T cell response at the clonal level simultaneously in peripheral blood samples and muscle biopsies. Previous studies using flow cytometry, immunochemistry and CDR3 spectra typing contributed to unraveling T cell involvement in myositis (11, 17) but are less sensitive and of low clonal resolution in detecting relevant repertoire changes (18).

In this prospective study on the effects of IVIG in IIM we explored T cell receptor beta (TCRβ) repertoires in paired peripheral blood samples and muscle biopsies taken prior to IVIG treatment. In this way we aimed to answer the question whether the peripheral blood T cell repertoire is reflective of the repertoire in muscle tissues prior to treatment. Our results confirmed T cell clonal expansion both in muscle tissues and in peripheral blood of IIM patients However, we also identified (expanded) tissue restricted TCRβ clones which could not be retrieved in peripheral blood. These tissue restricted TCRβ clones showed features which were quite distinct from those seen in peripheral blood, and shared between patients. Finally, TCRβ repertoire features in muscle tissues (but not in peripheral blood) correlated significantly with disease activity in these patients. Our data support a key role for abnormal T cell responses in disease development and progression in IIM.

## Materials and methods

### Ethical statement

All patients signed informed consent prior to inclusion in the study. This study was conducted with approval of the local medical research ethics committee of the Academic Medical Centre, Amsterdam, and in accordance with the declaration of Helsinki.

### Patients and study design

Peripheral blood and muscle tissues used in this study were baseline samples collected from adult, treatment-naive patients with newly diagnosed, biopsy-proven IIM included in a 9-week phase-2 open-label study that investigated intravenous immunoglobulins (IVIg) as first-line treatment in IIM patients (19). Two patients received immunosuppressants prior to collection of baseline samples but had no clinical benefits at time of enrolment in the study. For comparison, 10 healthy controls were included who did not have any disease nor drug use. Patient characteristics are summarized in **Table 1**.

**Table 1:** Characteristic of all 20 patients and healthy controls included in the study.

*Characteristic*	*Patients (n=20)*	*Healthy controls (n=10)*
Age at onset in years, median (IQR)	58 (40 – 69)	35 (30-55)
Months between start of symptoms until diagnosis, median (IQR)	5 (3-6)	0
Gender, females, n (%)	12 (60)	7 (70)
Connective tissue disorder, n (%)^¤^	3 (15)	0
Cancer, n (%)	1 (5)	0
Serum CK, median (IQR), U/L	1140 (182–5669)	NA
Myositis-specific and myositis associated antibodies Anti-HMGCR Anti-NXP2 Anti-Jo1 Anti-Mi2 Anti-MDA5 Anti-SRP Anti-TIF1gamma Myositis associated antibodies (MAA) only Seronegative	n (%) 3 (15) 3 (15) 1 (5) 1 (5) 1 (5) 1 (5) 1 (5) 2 (10) 7 (35)	N (%) 0 (0) 0 (0) 0 (0) 0 (0) 0 (0) 0 (0) 0 (0) 0 (0) 10 (100)
Myositis type DM IMNM NM/OM ASyS	n (%) 9 (45) 5 (25) 5 (25) 1 (5)	n (%) 0 (0) 0 (0) 0 (0) 0 (0)

IQR, interquartile range; CK, creatine kinase; U/L, units per litre. ^¤^ n=1 mixed connective tissue disease, n=1 Sjögren’s syndrome, n=1 systemic sclerosis; NA, not available; DM, Dermatomyositis; IMNM, Immune-mediated necrotizing myopathy; NM/OM, Non-specific myositis/overlapping myositis; ASyS, Anti-synthetase syndrome.

### Collection and processing of muscle tissues and blood samples

Muscle biopsies were taken upon diagnosis before treatment (baseline) in all patients. For collection of muscle biopsies, the optimal biopsy location was based on the presence of edema on muscle imaging (MRI or ultrasound). If no edema was present, the biopsy was taken from a clinically weak muscle. Biopsies were taken according to recommended standards for muscle biopsies (20). Muscle biopsies (stored in -80°C) were homogenized by spinning twice with 900µl of RLT buffer (1% B-mercaptoethanol) and ceramic beats in a MagNA Lyser (Roche) at 6500rpm for 30 seconds with cooling on ice for 1 minute in-between. The cell lysate was incubated with 60µl of Proteinase K and 240µl RNase free water for 10 minutes at 55°C on a shaking heat block. RNA was extracted using the RNeasy Mini kit (Qiagen, Hilden, Germany). Peripheral blood was drawn from all patients at baseline before start of treatment. Blood samples were all stored at -80°C until RNA isolation.

### Next-generation sequencing and bioinformatics analysis

Quantitative fingerprinting of lymphocyte repertoires was performed using Next-Generation Sequencing (NGS) as described earlier (21). In short, specific complementary DNA (specific-cDNA) of TCRβ molecules was synthesized using a TCRβ-chain Constant region reverse primer tagged with a 9 random nucleotide UMI and a consensus sequence. After specific-cDNA synthesis, Exonuclease I (Thermo Fisher Scientific, Breda, The Netherlands) treatment was performed to remove left over primers, followed by a multiplexed PCR with 23 forward primers covering all TCR β-chain Variable genes and a reverse primer binding to the consensus sequence previously introduced in the specific-cDNA and tagged with an 8 bp patient identifier (MID, Molecular Identifier). Obtained amplicons were purified using two rounds of AMPure XP beads clean-up (Beckman Coulter, Woerden, The Netherlands), quantified using Qubit dsDNA HS Assay Kit (Thermo Fisher Scientific), dual-indexed with i5 and i7 adapters (Nextera XT Index Kit v2) and sequenced using the Illumina Miseq Kit v3 2 x 300 bp technology according to the manufacturer’s manual (Illumina, San Diego, California, USA).

### TCR repertoire analysis

The list of final clones generated by RESEDA was analyzed using in-house developed scripts in R studio (R version 3.3.2) and further explained in the online supplementary materials and methods. The absolute number of mRNA molecules was indicated by the number of unique UMIs, hereafter referred to as reads. For analysis, 10,000 reads were randomly selected from each sample. The frequency of each clone was calculated as percentage of the total number of UMIs. Clones with a frequency ≥0.5% were defined as dominant clones based on previous observations (22). We defined the impact of a clone as its frequency in the repertoire, and the impact of a group of clones as their cumulative frequency. The total number of clones retrieved from sequencing are listed in **Supplementary Table 1**.

### Clustering related clones

Clones were grouped on the CDR3 amino acid sequence using in-house developed R scripts. In brief, the Hamming distance between clones was calculated. An approach similar to (23) was taken to calculate a dynamic threshold for grouping of clones. For every sample a density function was calculated over the Hamming distance matrix. This resulted in a bimodal distribution. The minimum between the two peaks was taken as a threshold with a minimum value of 3 amino acids. Clones with a Hamming distance below the dynamic threshold were grouped together to form a cluster and therefore considered to be related to each other.

### Evaluation of diversity and heterogeneity of TCRβ repertoires

To evaluate the diversity between the repertoires, the Shannon entropy, Simpson’s index and the Gini index were used. The Shannon entropy and Simpson’s index are able to measure the diversity in the TCRβ repertoires with a larger score indicating a higher diversity. Here the Shannon entropy is more appropriate to represent repertoire diversity within high-frequency reads, while the Simpson index is more sensitive to low-frequency reads. The Gini index is able to measure overall clonal expansion in a sample; it is bound between 0 and 1. A Gini index of 0 indicates that all the clones in the samples have the same frequency (i.e., all clones have equal number of clustered reads), whereas an index of 1 indicates that one single clone is responsible for all reads.

### Data analysis and statistics

Values are expressed as median and interquartile range, according to criteria for non-parametric analysis. Differences between groups were analyzed using Kruskal-Wallis or Mann Whitney non-parametric tests. Pearson’s correlation was used for correlation analysis. GraphPad Prism version 9.00 for Windows (GraphPad Software, La Jolla, California, USA) was used to perform the analyses. P-values < 0.05 were considered statistically significant.

## Results

### Patients

Twenty newly diagnosed treatment naïve IIM patients participated in the IMMEDIATE study, and outcomes have been reported previously (19). Nine (45%) patients had DM, five (25%) had NM/OM, five (25%) had IMNM and one patient (5%) had ASyS. No patients with IBM were included. All patients had proximal muscle weakness at inclusion. Muscle biopsies were taken from the vastus lateralis muscle in 16 patients, from a triceps muscle in two patients and from a deltoid muscle in two patients. All biopsied muscles showed edema on magnetic resonance imaging T2-weighed Dixon scans. For comparison, 10 healthy controls were included who did not have any neuromuscular or other known diseases nor drug use. Patient characteristics are summarised in **Table 1** below.

### Muscle tissues from myositis patients harbor expanded TCRβ clones

**Figure 1:**
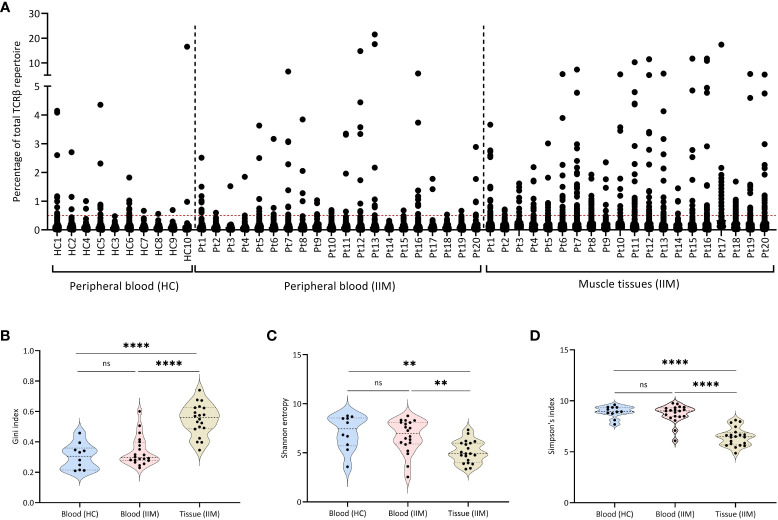
Expanded TCRβ clones in muscle tissues and peripheral blood. **(A)** Scatter plot of the TCRβ repertoire in muscle tissues and peripheral blood of treatment-naive IIM patients. Each dot represents a unique TCRβ clone. The dotted line on the Y-axis (0.5%), represents the cut-of used to label clones are dominant. **(B)** Gini index, **(C)** Shannon entropy and **(D)** Simpson index for the TCRβ repertoires in peripheral blood of healthy controls (HC) and of IIM patients, and in muscle tissues of IIM patients. ns = not significant (p>0.05), **p<0.001, ****p<0.0001.

To better understand T cell responses at the clonal level in muscle tissues and peripheral blood of IIMs patients, we sequenced the TCRβ chain repertoire in muscle biopsies and peripheral blood of all 20 patients included in the IMMEDIATE study (19) and compared the results to healthy control peripheral blood TCRβ repertoires. In muscle tissues and peripheral blood of all 20 IIM patients as well as in peripheral blood of some healthy controls, we detected multiple expanded TCRβ clones (**Figure 1A**).

Compared to the blood samples of healthy control and IIMs patients, the TCRβ repertoires from muscle tissues of IIM patients showed significantly more expanded TCRβ clones, as shown by a significantly higher Gini index (**Figure 1B**), also reflected in a significantly lower diversity both in the Shannon entropy (**Figure 1C**) and in Simpson’s index (**Figure 1D**). Grouping of patients on myositis subtype or on the presence of different antibodies (myositis-specific, myositis-associated and seronegative) did not reveal differences in the TCRβ repertoires (**Supplementary Figure 1**). At baseline in blood or muscle tissue the number of dominant BCRH clones assessed previously in this cohort (9) did not correlate with the number of dominant TCRβ clones present in each patient.

In summary, muscle tissues of IIM patients show more expanded clones and a less diverse TCRβ repertoire when compared to the repertoires seen in peripheral blood of these patients as well as in peripheral blood of healthy controls.

### Dominant clones are shared between muscle and blood

**Figure 2:**
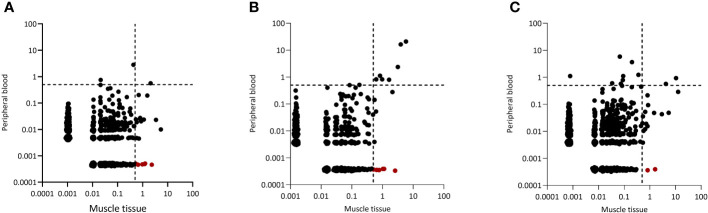
Representative examples of CDR3 clonal overlap between peripheral blood and muscle tissue. Plots are shown for **(A)** one dermatomyositis (DM) patient, **(B)** one IMNM patient and **(C)** one NM/OM patient. Each dot represents a unique TCRβ clone, and its frequency in the analyzed repertoires is depicted on the x (muscle tissue) and y (peripheral blood) axes as percentage of total UMIs. The dotted lines indicate the 0.5% cut-off for dominant TCRβ clones. TCRβ clones in red are clones which are tissue restricted and not found in peripheral blood.

Since dominant clones were present in both blood and muscle tissue, we wondered whether peripheral blood and muscle tissue shared the same dominant clones. Indeed, in all 20 IIM patients some dominant clones found in muscle tissues could be readily retrieved in peripheral blood and in 13 of the 20 patients analyzed, some of these clones even remained dominant in the peripheral blood TCRβ repertoire. Interestingly, of the 352 dominant clones detected in muscle tissues of all 20 patients, 97 (28%) of these clones were muscle-restricted as they could not be retraced in the paired peripheral blood TCRβ repertoire (**Figures 2A–C**, tissue restricted dominant clones colored in red and **Supplementary Figure 2** for the remaining patients).

### Characteristics of muscle and peripheral blood TCRβ clones

**Figure 3:**
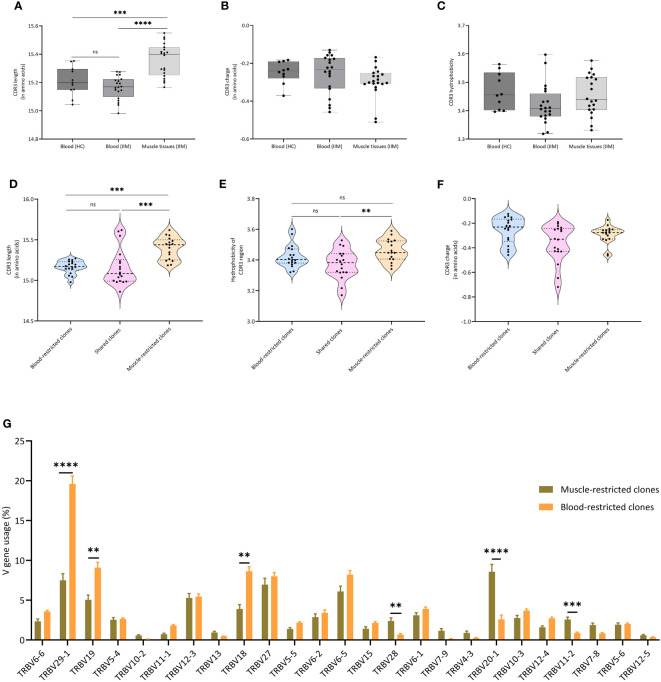
Characteristics of TCRβ clones in muscle and blood **(A)** Average CDR3 length, **(B)** Average CDR3 charge and **(C)** Average hydrophobicity of clones in peripheral blood of healthy controls, and in peripheral blood and muscle tissues of IIM patients. ***p<0.0007, ****p<0.0001. **(D)** CDR3 length, **(E)** CDR3 charge and **(F)** CDR3 hydrophobicity of clones found exclusively in muscle tissues (Muscle-restricted clones), clones shared between muscle tissues and blood (Shared clones) and clones found exclusively in blood (Blood restricted clones). **(G)** V gene usage of all clones as percentage of the TCRβ repertoire. Data are presented as median and IQR and analyzed using two-way ANOVA. To correct for multiple testing, the Bonferroni’s multiple comparison test was used. **<0.001, ***p<0.0002, ****p<0.0001.

A recent report indicated that amino acid composition, charge, hydrophobicity, length of the CDR3 region and V gene usage are crucial in determining the phenotype and fate of T cells (24). We compared these properties of the clones in our peripheral blood and muscle samples. While charge and hydrophobicity did not differ significantly, clones from muscle tissue had significantly longer TCRβ CDR3s compared to clones in peripheral blood of IIM patients (p=0.0001) or healthy controls (p=0.0007) (**Figures 3A–C**). Furthermore, clones in muscle tissue showed significantly increased usage of certain TCRVβ genes, notably of TRBV20-1. In peripheral blood TRBV29-1 was the most used V gene, both in IIM patients and in healthy controls (**Supplementary Figures 3A, 2B**). Next, to investigate whether similar TCRβ clones could be found in muscle tissues of different myositis patients, we performed a clustering analysis at the CDR3 level of all dominant clones found in muscle tissues. Indeed, TCRβ clones from different patients clustered together based on CDR3 amino acid similarity (**Supplementary Figure 4**; **Supplementary Table 2**).

When we compared muscle-restricted clones to clones shared between peripheral blood and muscle tissue, CDR3 charge did not differ. However, we observed that muscle-restricted clones had significantly longer CDR3 regions than shared (p=0.0002) or blood-restricted clones (p=0.001) and encoded more hydrophobic CDR3 regions than blood-restricted clones (p=0.009) (**Figures 3D–F**). Also, comparison analysis of the V-gene usage showed a significantly increased frequency of TRBV20-1 in muscle-restricted clones (p<0.0001), while TRBV29-1 was significantly increased in blood-restricted clones (p<0.0001) (**Figure 3G**).

In summary, muscle tissues and peripheral blood of IIM patients share dominant TCRβ clones. TCRβ clones present in muscle, and especially the muscle-restricted clones, have features which are distinct from those seen in clones found exclusively in peripheral blood and in clones shared between muscle tissues and peripheral blood.

### Correlation of TCRβ diversity in muscle tissues and peripheral blood with disease activity

**Figure 4:**
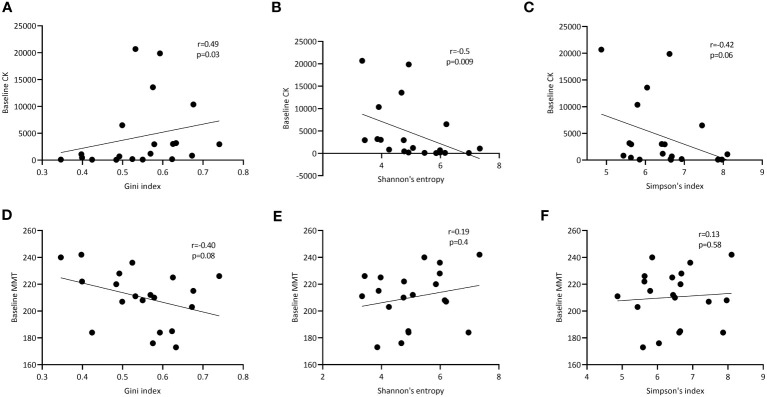
Correlation of TCRβ diversity and heterogeneity in muscle tissues with markers of disease activity. Correlation of respectively the Gini, Shannon entropy and Simpson index of the TCRβ repertoire in muscle tissue with CK levels at baseline **(A-C)** and manual muscle testing (MMT; **D-F**). p- and R-values are shown for each curve.

Finally, we assessed whether TCRβ repertoire characteristics associated with markers of disease activity. In peripheral blood, we did not see any significant correlations between clonality (number and impact of dominant clones) as well as diversity and heterogeneity of the TCRβ repertoire with muscle strength (MMT), creatine kinase levels (CK) and muscle imaging (data not shown). However, in muscle tissue the Gini index correlated significantly with the creatine kinase (CK) levels (r=0.49; p=0.03; **Figure 4A**) and trended to correlate inversely with muscle strength (MMT; r=-0.40; p=0.08; **Figure 4D**). CK was negatively correlated with repertoire diversity as assessed by the Shannon entropy (r=-0.50, p=0.009) and Simpson’s index (r=-0.42, p=0.06; **Figures 4B, C**). Diversity was not correlated with MMT (**Figures 4E, F**).

In conclusion, while various features of the TCRβ repertoire in blood do not correlate with markers of disease activity, in muscle tissue the Gini index, a measure of clonal expansion within the TCRβ repertoire correlates with CK levels, while TCRβ diversity inversely correlates with CK.

## Discussion

Using a highly sensitive UMI-based method for TCRβ sequencing, we showed that muscle tissues and peripheral blood of IIM patients harbor expanded TCRβ clones. Furthermore, the TCRβ repertoire in muscle tissues is not fully reflective of the TCRβ repertoire in peripheral blood. We identified multiple tissue restricted clones which possessed properties which were different from TCRβ clones that are (also) found in circulation. Additionally, the TCRβ repertoire in muscle tissue shows a higher impact of expanded clones and is less diverse compared to the repertoire in peripheral blood. This increased clonal expansion and decreased diversity in muscle tissue correlates significantly with increased disease activity as measured by CK.

The presence of autoantibodies in a group of IIM patients suggests that components of the adaptive immune response play a role in the pathogenesis of the disease, which also includes T cells (15, 16, 25). While flow cytometric tools have been instrumental in unravelling T cell responses and phenotypes in IIMs, TCR sequencing has emerged as a powerful tool to examine the T cell repertoire in more detail (22, 26). Our results are in line with previous reports that have described clonal expansion of T cells in dermatomyositis (11, 12, 27). In addition, our report on the presence of expanded clones in immune-mediated necrotizing myopathy as well as anti-synthetase syndrome is in line with a recent study that reported the presence of expanded T cell clones in IMNM and ASyS patients (13). We would like to note that sequencing alone does not yield information on the specificity and pathogenicity of clones. This makes it difficult to determine the pathologic relevance of these expanded clones, and we cannot exclude that some of these clones might be mere bystanders. Since we previously showed clonal expansion of B-cell receptor clones in muscle and blood of myositis patients in this same cohort (9), our analysis on whether the individuals with the most expanded BCRH clones where the same individuals with the most expanded TCRβ clones did not yield fruits as we did not observe any significant correlations. A possible explanation is the phenotype of B and T cells in play.

Since muscle is one of the most affected tissues in myositis, an intriguing question is whether the peripheral blood TCRβ repertoire is reflective of the muscle TCRβ repertoire. While we could retrieve most expanded blood T cell clones in muscle tissues, 28% of the dominant muscle infiltrating expanded clones could not be found in blood. This indicates that some T cells present in muscle tissue do not recirculate into the periphery (12, 17), or alternatively are present in blood in lower frequencies below the detection limit of our repertoire analysis. These clones might represent tissue-resident T cells or T cells recruited from the periphery and primed in the muscle. A prime candidate for such T cells are cytotoxic T cells which might express homing receptors which allow their firm attachment to muscles. These cells produce granzyme and perforins which might subsequently result in muscle injury and weakness (28). Future work tailored towards unravelling the phenotype and pathogenicity of these tissue restricted clones will be of importance.

We also observed that the muscle TCRβ repertoires were less diverse and more expanded than the repertoires in peripheral blood and in healthy controls. These results suggest the presence of an antigen-driven immune response in muscle tissues of myositis patients. These results are in accordance with data from other autoimmune disorders that have reported a lower diversity of the TCR repertoires in these diseases compared to that in healthy controls. Such a decrease in TCR diversity has been proposed to be a characteristic feature of autoimmune T cell repertoires (29–31). Interestingly in systemic sclerosis patients undergoing autologous hematopoietic stem cell transplant, non-responders were shown to have a less diverse TCR repertoire after treatment (32), further providing evidence that a decrease TCR repertoire might contribute to the pathogenesis of autoimmune disorders.

Next, when allowing for up to 3 mismatches in the CDR3 region, we could identify similar and shared dominant clones between different individuals. The presence of such similar T cell responses might indicate the presence of responses against similar antigens in muscle tissues of different myositis patients. However, future work tailored towards unravelling the specificity of these similar T cell clones is crucial as this may result in the identification of common disease pathways that may be targeted therapeutically.

When looking at the properties of the identified CDR3 sequences, we observed that the TCRβ sequences which were present exclusively in muscle tissues were much longer that the sequences observed in blood of myositis patients. Previous studies in B-cells showed that longer CDR3 loops are associated with auto-reactivity. Healthy control repertoires were mostly composed of shorter CDR3 sequences, which might be due to efficient removal of BCRs with longer CDR3 sequences (33, 34). A similar selection of shorter CDR3 TCR sequences occurs in the thymus (35). Therefore, it is tempting to speculate that the longer CDR3 TCRβ sequences in our muscle repertoires could bear auto-reactive properties. In addition, we also observed that the TCRβ clones showed a preferential usage of certain V genes, TRBV20-1 being the most dominantly used V gene in clones found exclusively in muscle tissues, and TRBV29 gene being the most dominantly used V gene segment in clones found exclusively in blood. This predominance of TRBV20-1 could be due to an antigen-driven immune response especially in the muscle microenvironment. Interestingly, a recent study also reported preferential usage of the TRBV20-1 gene by citrulline specific T cell clones in rheumatoid arthritis synovial fluid, a phenomenon which was not seen in influenza-specific T cell clones (36). Such a bias in V gene usage further strengthens the notion that this preferential usage of specific V genes could be antigen-driven. Therefore, future studies in TCR transgenic mice could fully unravel the potential pathogenicity of clones that bear such V genes as well as the potential to target them therapeutically.

Finally, an interesting question is whether the TCRβ repertoire in both muscle tissues and peripheral blood of IIM patients is associated with disease activity. While we did not see any significant correlations between TCRβ clonality in blood with disease activity markers, we noted that increased expansion of TCRβ clones in muscle tissue was associated with higher creatine kinase levels and – non-significantly (p=0.08) - with decreased muscle strength. These results suggest that abnormal clonal expansion in muscle tissues might be a player in the pathogenesis of this disease, but these observations will need confirmation.

Our study has several limitations. First, the relatively low number of patients included in the study did not allow extensive subgroup analysis for different myositis subtypes. Secondly, our analysis yields little information on the phenotype of muscle-restricted and of circulating dominant TCRβ clones. As a result, extensive approaches such as cell stimulation assays as well as antigen specific cell sorting are needed to fully unravel the specificity and pathogenicity of the dominant TCRβ clones identified. Nevertheless, our findings on distinct features of tissue restricted clones as well as a correlation between the TCRβ diversity and disease activity may point to a pathogenic role of these clones in the pathogenesis of the disease. Another limitation is that our repertoire analysis was performed on the RNA level. This implies that the observed TCR clonality differential TCR expression per cell type, e.g. based on activation or differentiation status, might influence our frequency estimates. However, studies indicate that on the genomic level this phenomenon is not as profound as observed in the B-cell lineage (e.g. comparing naïve B-cells and plasma cells) (37).

In summary, we have shown the presence of highly expanded TCRβ clones in muscle tissues and peripheral blood of IIM patients. Network analysis shows clonal motifs are shared between different patients. We identified muscle-specific TCRβ clones whose receptors show specific characteristics, including increased CDR3-length, specific V-gene usage and increased CDR3-hydrophobicity. Finally, increased clonal expansion and decreased diversity of the TCRβ repertoire in muscle tissue correlated with increased disease activity as measured by increased CK. If confirmed the latter observation may point to a role for dominant muscle clones in disease pathogenesis.

## Data availability statement

The dataset generated and presented in this study can be retrieved online. The repository and accession number can be found in the following link: https://www.ncbi.nlm.nih.gov/PRJNA941242. All scripts used to analyse data are available from the corresponding author on reasonable request.

## Ethics statement

The studies involving humans were approved by Local medical research ethics committee of the Academic Medical Centre, Amsterdam. The studies were conducted in accordance with the local legislation and institutional requirements. The participants provided their written informed consent to participate in this study.

## Author contributions

DA: Formal Analysis, Writing – original draft, Data curation, Investigation, Software, Validation, Visualization. HW: Formal Analysis, Investigation, Writing – original draft, Methodology. JL: Conceptualization, Investigation, Methodology, Writing – original draft. IN: Investigation, Visualization, Writing – original draft. LV: Methodology, Validation, Visualization, Writing – original draft. EA: Investigation, Methodology, Writing – original draft. FE: Investigation, Writing – original draft, Formal Analysis, Methodology. JR: Conceptualization, Funding acquisition, Supervision, Writing – original draft. BV: Data curation, Software, Writing – review & editing. AV: Software, Supervision, Writing – original draft, Methodology. AK: Conceptualization, Funding acquisition, Supervision, Writing – original draft, Investigation, Methodology. ND: Formal Analysis, Funding acquisition, Investigation, Methodology, Supervision, Visualization, Writing – original draft.
