# Supplementary material for: TCRβ clones in muscle tissue share structural features in patients with idiopathic inflammatory myopathy and are associated with disease activity
Source: Front Immunol. 2024 Jan 10;14:1279055. doi: 10.3389/fimmu.2023.1279055 (PMC10806010; doi:10.3389/fimmu.2023.1279055)
Supplement: Supplementary file 1 [file DataSheet_1.docx]

**Supplementary Table 1: Number of TCRβ sequences (clones) obtained from all patients.**

| **Patient** | **Number of sequences in blood** | **Number of sequences in muscle tissue** |
| --- | --- | --- |
| **Pt1** | 8958 | 1697 |
| **Pt2** | 10787 | 5045 |
| **Pt3** | 21743 | 462 |
| **Pt4** | 23252 | 1949 |
| **Pt5** | 16584 | 7407 |
| **Pt6** | 14064 | 2609 |
| **Pt7** | 11721 | 774 |
| **Pt8** | 13088 | 1369 |
| **Pt9** | 8543 | 3416 |
| **Pt10** | 16204 | 446 |
| **Pt11** | 9514 | 1763 |
| **Pt12** | 8056 | 980 |
| **Pt13** | 10410 | 1996 |
| **Pt14** | 13039 | 4024 |
| **Pt15** | 16392 | 2770 |
| **Pt16** | 14091 | 2578 |
| **Pt17** | 13269 | 313 |
| **Pt18** | 10392 | 1367 |
| **Pt19** | 17143 | 2140 |
| **Pt20** | 7058 | 1107 |

**Supplementary Figure 1:** Clonality (the number and impact of dominant clones) and diversity of the TCRβ repertoires in blood and muscle tissues of the different myositis subtypes (DM, IMNM, NM/OM and ASyS) and different antibody groups (MSA, MAA and seronegative). DM; dermatomyositis, IMNM; immune-mediated necrotizing myopathy, NM/OM; non-specific/overlapping myositis, ASyS; anti-synthetase syndrome, MSA; myositis-specific antibody, MAA; myositis-associated antibody

**
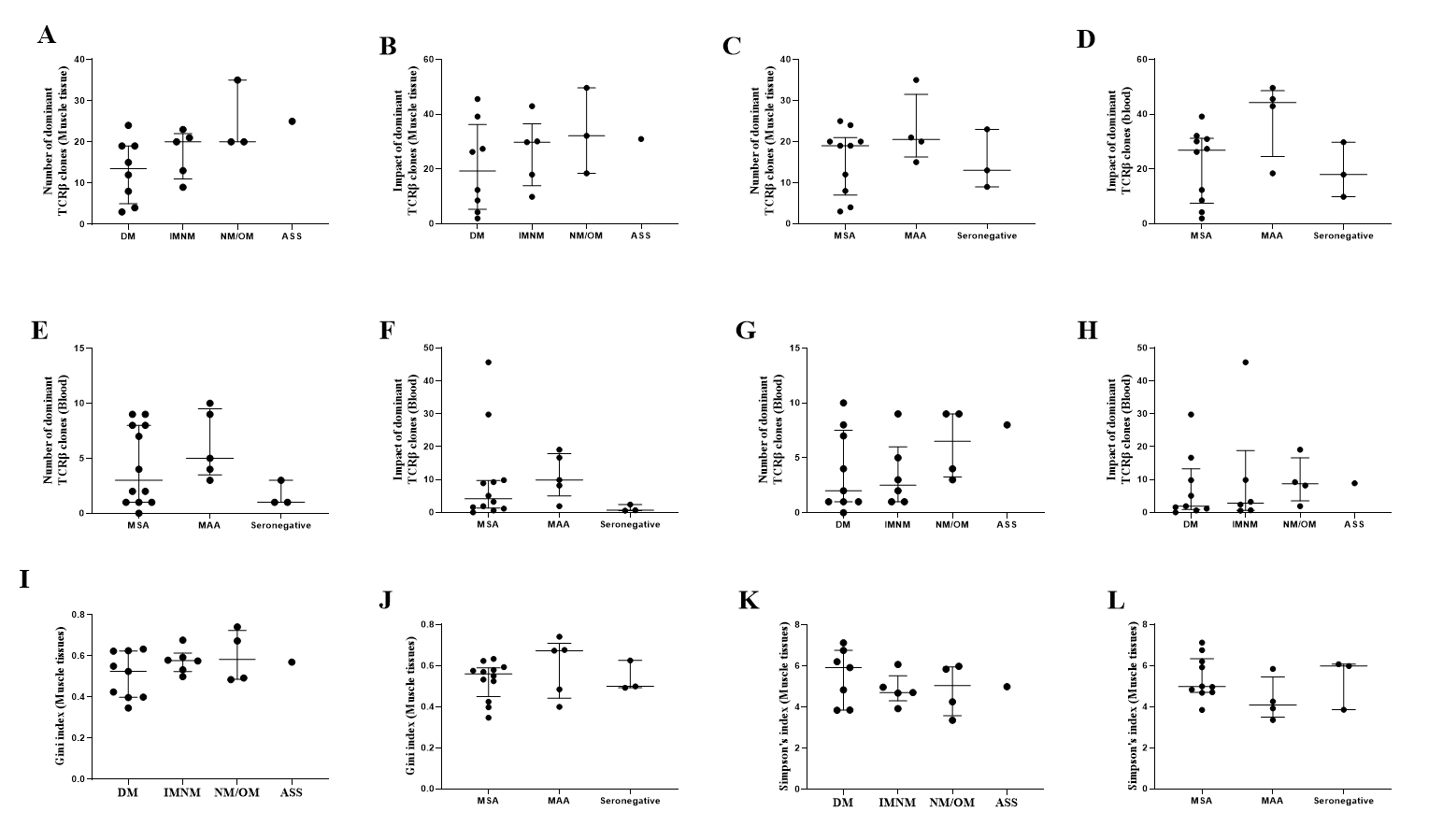
**

**Supplementary figure 2** : CDR3 overlap plots of muscle tissues and peripheral blood for the remaining 17 patients is shown. Each dot represents a unique TCRβ clone, and its frequency in the analyzed repertoires is depicted on the x (muscle tissue) and y (peripheral blood) axes as percentage of total UMIs. The dotted lines on each axis indicate the 0.5% cut-off for dominant TCRβ clones.


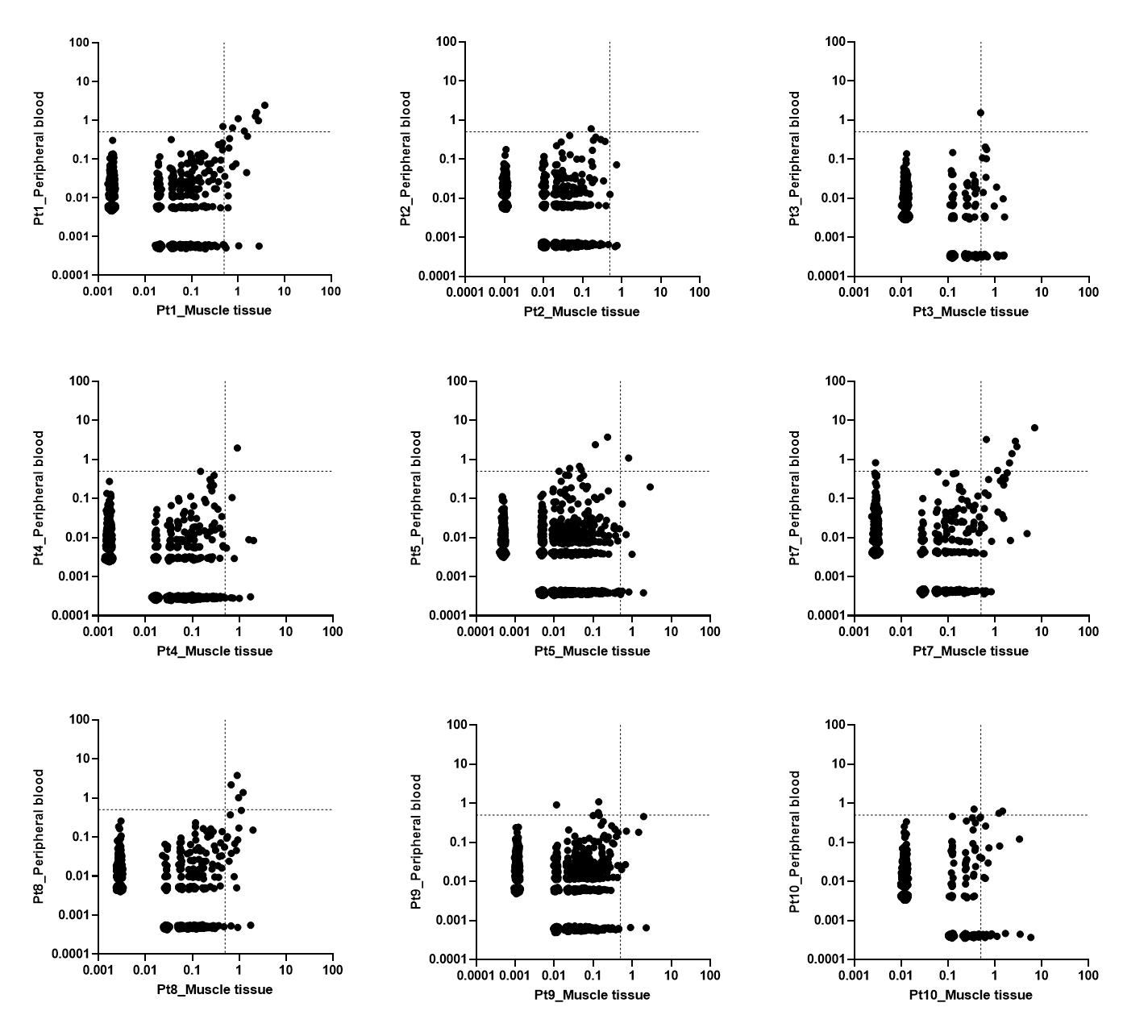


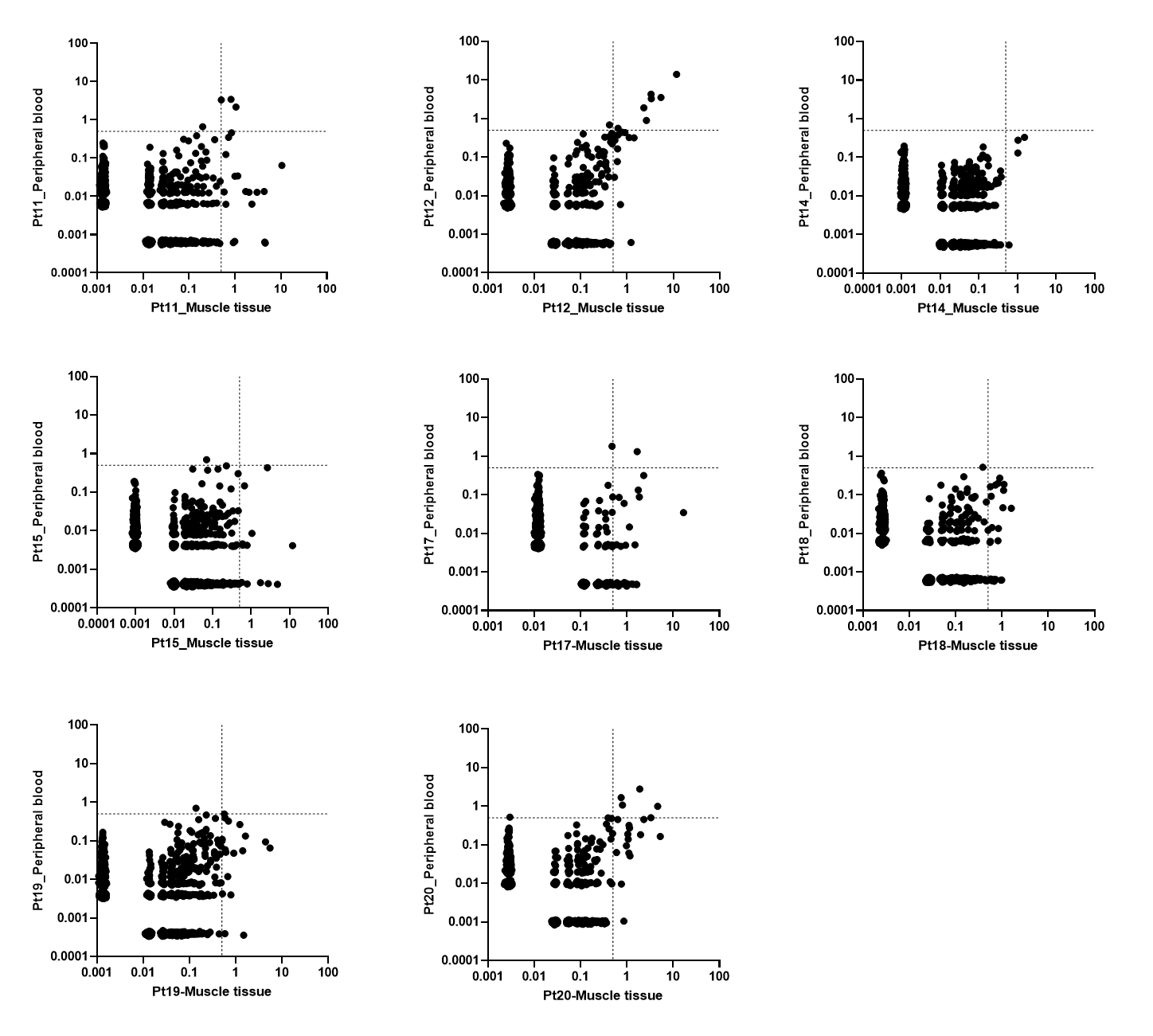


**Supplementary figure 3:** V and J gene usage of TCRβ clones present in muscle tissues and peripheral blood of IIM patients.

**Supplementary figure 4**: CDR3 cluster analysis of all dominant TCRβ clones detected in **(A)** muscle tissue and **(B)** Peripheral blood of all 20 IIM patients. Each dot represents a unique CDR3 dominant TCRβ clone. Clones of the same color are dominant clones which originate from the same patient. The lines connect clones which are similar to each other at the CDR3 amino acid level. Clones were considered to be similar if they differed by ≤ 3 amino acids in the CDR3 region


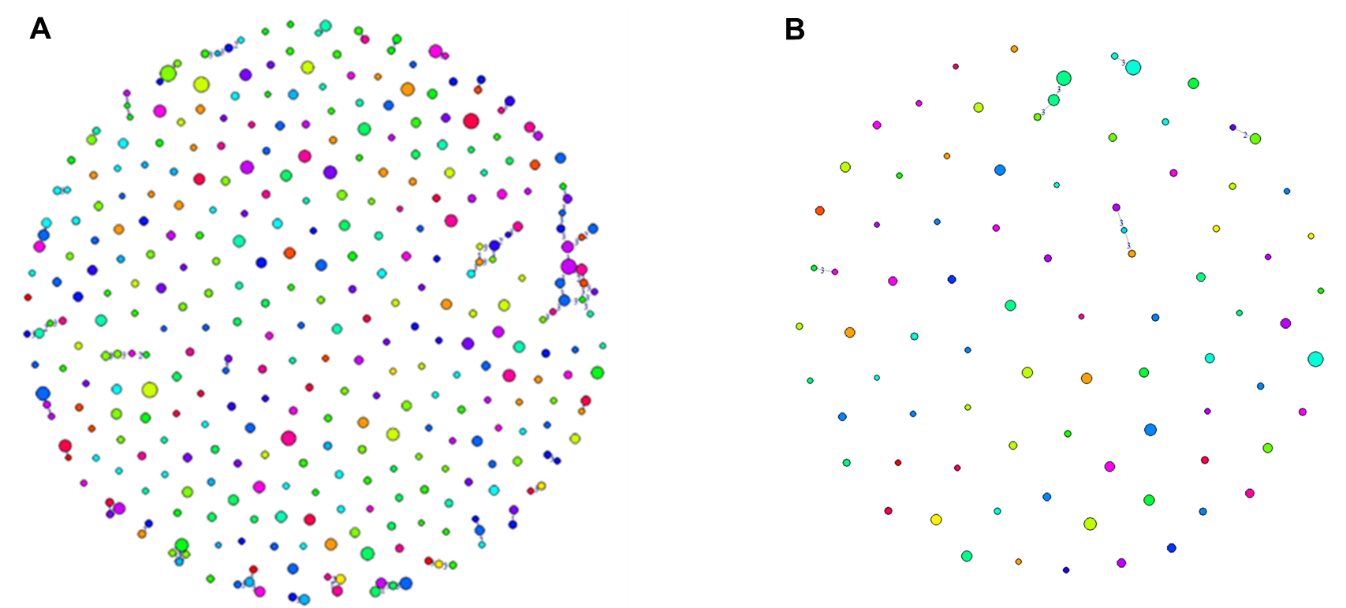


**Supplementary table 2**: List of CDR3 amino acid sequences from clusters in the network analysis

| **Cluster**  **number** | **Patient ID** | **CDR3 sequence** | **Frequency** | **V gene** | **J gene** |
| --- | --- | --- | --- | --- | --- |
| 6 | Pt10 | CASRGELNTEAFFG  CASSLDQVNEQFFG | 1.2  0.6 | TRBV5-4  TRBV11-2 | TRBJ1-1  TRBJ2-1 |
|  | Pt19 | CASRIDGNTEAFFG  CASSLTSSNEQFFG | 0.6  0.6 | TRBV12-3  TRBV27 | TRBJ1-1  TRBJ2-1 |
|  | Pt1 | CASSETAGNEQFFG | 0.6 | TRBV6-5 | TRBJ2-1 |
|  | Pt17 | CASSLAGTPEAFFG | 0.6 | TRBV7-9 | TRBJ1-1 |
|  | Pt11 | CASSLGDTDEAFFG  CASSWAGQNEQFFG | 2.9  0.6 | TRBV5-6  TRBV11-2 | TRBJ1-1  TRBJ2-1 |
|  | Pt7 | CASSLGLSDEQFFG  CASSLYLNTEAFFG  CASSQGLYNEQFFG  CASSQTGSTEAFFG | 2.8  0.6  1.4  1.6 | TRBV12-3  TRBV27  TRBV27  TRBV4-1 | TRBJ2-1  TRBJ1-1  TRBJ2-1  TRBJ1-1 |
|  | Pt5 | CASSRTGDTEAFFG  CASSWDSDNEQFFG | 0.6  0.9 | TRBV11-1  TRBV11-1 | TRBJ1-1  TRBJ2-1 |
|  | Pt12 | CASSWGTDNEQFFG  CASSWGVDTEAFFG | 11.5  3.4 | TRBV6-5  TRBV6-5 | TRBJ2-1  TRBJ1-1 |
| 62 | Pt14 | CASSAHRDTQYFG | 0.9 | TRBV28 | TRBJ2-7 |
|  | Pt2 | CASSLGRGTQYFG | 0.7 | TRBV11-1 | TRBJ2-5 |
|  | Pt18 | CASSPQSDTQYFG | 0.8 | TRBV6-2 | TRBJ2-3 |
| 146 | Pt13 | CASSESGHRNQPQHFG | 2.1 | TRBV10.2 | TRBJ1-5 |
|  | Pt4 | CASSLSGKSNQPQHFG | 1.7 | TRBV27 | TRBJ1-5 |
|  | Pt2 | CASSLWGEGNQPQHFG | 0,7 | TRBV5-5 | TRBJ1-5 |
|  | Pt7 | CASSRRGGSNQPQHFG | 0.7 | TRBV27 | TRBJ1-5 |
| 10 | Pt2 | CASSAAGASTDTQYFG | 0.5 | TRBV6.5 | TRBJ2-3 |
|  | Pt15 | CASSKAHDSTDTQYFG | 4.9 | TRBV21-1 | TRBJ2-3 |
| 122 | Pt18 | CSARAGETQYFG | 0.6 | TRBV20-1 | TRBJ2-5 |
|  | Pt4 | CSARGSGGGVYNEQFFG | 1.3 | TRBV20-1 | TRBJ2-1 |
|  | Pt8 | CSARLPDTQYFG | 1.7 | TRBV20-1 | TRBJ2-3 |
|  | Pt16 | CSARSGNTIYFG | 0.7 | TRBV20-1 | TRBJ1-3 |
|  | Pt10 | CSASGVDTQYFG | 1.1 | TRBV20-1 | TRBJ2-3 |
| 25 | Pt8 | CASSFWGGQETQYFG | 1.0 | TRBV11.2 | TRBJ2-5 |
|  | Pt5 | CASSPLGHQETQYFG | 0.8 | TRBV27 | TRBJ2-5 |
| 23 | Pt11 | CASSLQGVNQPQHFG | 1.6 | TRBV27 | TRBJ1-5 |
|  | Pt9 | CASSPTGGGQPQHFG  CASSPTGNNQPQHFG | 2.3  0.5 | TRBV12-3  TRBV12-3 | TRBJ1-5  TRBJ1-5 |
|  | Pt17 | CASSQSQGGQPQHFG | 0.6 | TRBV6-6 | TRBJ1-5 |
|  | Pt16 | CASSTLGKGQPQHFG | 0.6 | TRBV19 | TRBJ1-5 |
|  | Pt6 | CASSTLQGSQPQHFG | 0.8 | TRBV19 | TRBJ1-5 |
|  | Pt3 | CASSTNWGNQPQHFG | 1.0 | TRBV6-5 | TRBJ1-5 |
| 31 | Pt6 | CASRISNQPQHFG | 0.5 | TRBV6-2 | TRBJ1-5 |
|  | Pt15 | CASVQGNQPQHFG | 1.7 | TRBV19 | TRBJ1-5 |
| 33 | Pt20 | CASSGGQSSYNEQFFG  CASSLGQSSYNEQFFG | 1.9  1.1 | TRBV20-1  TRBV20-1 | TRBJ2-1  TRBJ2-1 |
|  | Pt7 | CASSLWGSPYNEQFFG | 4.7 | TRBV20-1 | TRBJ2-1 |
|  | Pt12 | CASSPLTSSYNEQFFG | 2.2 | TRBV7-8 | TRBJ2-1 |
| 49 | Pt13 | CASSLGDTTDTQYFG | 2.6 | TRBV7-2 | TRBJ2-3 |
|  | Pt7 | CASSLGGAGDTQYFG | 3.0 | TRBV12-3 | TRBJ2-3 |
|  | Pt3 | CASSPTGFGDTQYFG | 1.5 | TRBV6-5 | TRBJ2-3 |
| 52 | Pt3 | CASSLFQGSYGYTFG | 0.6 | TRBV20-1 | TRBJ1-2 |
|  | Pt8 | CASSLSGTDYGYTFG | 0.6 | TRBV20-1 | TRBJ1-2 |
|  | Pt7 | CASSLTGGSYGYTFG | 1.5 | TRBV20-1 | TRBJ1-2 |
| 56 | Pt8 | CASSLRHGLQPQHFG | 0.9 | TRBV20-1 | TRBJ1-5 |
|  | Pt4 | CASSYRAGHQPQHFG | 2.2 | TRBV6-2 | TRBJ1-5 |
| 62 | Pt12  Pt12 | CASSPTGPGTEAFFG  CASSPTGPWGEQFFG | 0.7  0.6 | TRBV20-1  TRBV6-5 | TRBJ1-1  TRBJ2-1 |
|  | Pt7 | CATSPRGVGTEAFFG | 7.3 | TRBV20-1 | TRBJ1-1 |
| 75 | Pt11 | CASRPGTGQPQHFG | 0.5 | TRBV20-1 | TRBJ1-5 |
|  | Pt9 | CASSPWDGQPQHFG | 1.8 | TRBV18 | TRBJ1-5 |
| 74 | Pt4 | CSARAGIVNTEAFFG | 0.5 | TRBV20-1 | TRBJ1-1 |
|  | Pt19 | CSARSGLMNTEAFFG | 0.7 | TRBV20-1 | TRBJ1-1 |
|  | Pt10 | CSARSRTGNTEAFFG | 0.9 | TRBV20-1 | TRBJ1-1 |
| 76 | Pt10 | CASSIGLPNTEAFFG | 0.7 | TRBV5-4 | TRBJ1-1 |
|  | Pt112 | CASSLGGSNTEAFFG | 3.4 | TRBV12-3 | TRBJ1-1 |
|  | Pt115 | CASSPGTANTEAFFG | 1.0 | TRBV27 | TRBJ1-1 |
| 104 | Pt12 | CASSPPATGYNEQFFG | 1.3 | TRBV5-4 | TRBJ2-1 |
|  | Pt11 | CASSPSRTGTNEQFFG | 1.9 | TRBV1-9 | TRBJ2-1 |
